# Introduction to the Rosetta Special Collection

**DOI:** 10.1371/journal.pone.0144326

**Published:** 2015-12-29

**Authors:** Sagar D. Khare, Timothy A. Whitehead

**Affiliations:** 1 Department of Chemistry and Chemical Biology, Rutgers University, Piscataway, NJ, United States of America; 2 Department of Chemical Engineering and Materials Science, Michigan State University, East Lansing, MI, United States of America; 3 Department of Biosystems and Agricultural Engineering, Michigan State University, East Lansing, MI, United States of America; University of Michigan, UNITED STATES

## Abstract

The Rosetta macromolecular modeling software is a versatile, rapidly developing set of tools that are now being routinely utilized to address state-of-the-art research challenges in academia and industrial research settings. A Rosetta Conference (RosettaCon) describing updates to the Rosetta source code is held annually. Every two years, a Rosetta Conference (RosettaCon) special collection describing the results presented at the annual conference by participating RosettaCommons labs is published by the Public Library of Science (PLOS). This is the introduction to the third RosettaCon 2014 Special Collection published by PLOS.

## Introduction

The Rosetta macromolecular modeling software is a versatile, rapidly developing set of tools that are now being routinely utilized to address state-of-the-art research challenges in academia and industrial research settings. The software is being co-developed by 44 laboratories from universities, government labs, and research centers in the United States, Europe, Asia, and Australia. The Rosetta software package is the result of a collaborative effort among these research institutions, building upon shared discoveries and free exchange of knowledge and software tools. Every institution with a participating laboratory is a member of an organization called RosettaCommons that facilitates code development and collaboration (http://www.rosettacommons.org). To enhance this collaborative development effort, RosettaCommons holds an annual conference in Leavenworth, WA, USA in the last week of July or the first week of August. Every two years, a Rosetta Conference (RosettaCon) special collection describing the results presented at the conference by participating RosettaCommons labs is published by the Public Library of Science (PLOS). As organizers of the 2014 Rosetta Conference, we are pleased to introduce the third RosettaCon 2014 Special Collection published by PLOS.

The applications of Rosetta software can be broadly divided into two themes–modeling or predicting structures of natural biological polymers [1,2], and the design of novel biomacromolecules [3,4] using, in some cases, an expanded alphabet that included non-natural sidechain and/or backbone functional groups [5,6]. These diverse applications, however, use the same underlying conceptual and software framework consisting of generating various conformations (sampling) of a molecule and scoring these conformations to identify optimal atomic-resolution arrangements (energy function). A crucial early insight was that both scoring and sampling techniques should ideally be independent of the problem under consideration and trained on experimental data [7]. Examples of these datasets include the distributions of protein backbone conformations or side chain rotamers seen in the Protein Databank [1,8], or the measured changes in free energies upon mutation in protein cores [9]. In this framework, the successes and failures of each structural modeling or design exercise provides valuable feedback for improving the underlying methods to iteratively recapitulate a greater proportion of experimental results. Therefore, reproducibility, verification and generalizability of new Rosetta computational algorithms is crucial.

A recent report extrapolates that fully 50% of biological research is not reproducible [10]. Accessibility of new techniques to an outside user can significantly impact reproducibility [11]. In principle, computational biology simulations should offer greater control over both accessibility and reproducibility compared to “wet” lab experiments, as the number of uncontrolled ingredients (reagents etc.) are lower. Yet in practice both reproducibility and accessibility can suffer. This is because academic labs often develop shortcuts and shorthand in day-to-day practice of a newly developed technique, and often omit to mention these little details in their publications, which, in turn, may contribute negatively to reproducibility. Additionally, the structural and design complexity of multi-purpose software such as Rosetta is high (currently at 2.7 million lines of code) and new software developments are usually made in academic laboratories by non-professional software developers who are focused on solving a specific *scientific* problem. For example, the use of specific data structures that assume molecular connectivity corresponding to canonical L-amino acids can frustrate the extension of a structure prediction algorithm to non-canonical side chains or backbone groups.

One idea to achieve reproducibility and accessibility was explored in the previous Rosetta collections—Protocol Capture [12]. In a Protocol Capture, all individual steps in a newly developed protocol are listed as a step-by-step flowchart [13]. Input and expected output files, along with a reference to the code executable (or version number), are provided to the user. In this manner, the user can identify what was actually done in the simulation. This helps both scientific reproducibility (by reporting exactly what was done) as well as accessibility (by allowing non-specialists to reproduce the main findings of the work). However, the issues of laboratories using their shorthand and assumptions, as well as insufficient attention being paid to generalizability still remained. In this collection, we sought to address these issues by requiring an author from an external (but still RosettaCommons) laboratory to serve as a “tester”. This follows from the well-established practice in the software industry where testing and development are separate functions. For the Rosetta community, this approach provides the additional benefit that the external “tester” author, while being an expert in the general area, is sufficiently removed from the laboratory-specific jargon and project-specific scientific goals. Thus, the perspective of the tester author should increase the clarity of description as well as generalizability of the underlying code itself.

This year’s collection contains 12 papers published in PLOS One and PLOS Computational Biology. These papers characterize the diversity of modeling applications present in the Rosetta Macromolecular Code framework, including structure prediction, protein design, modeling of conformational states, and enzyme redesign. We have grouped the papers into four broad categories: structure prediction, membrane proteins, scientific benchmarks, and docking. Many of these categories are artificial, as some of the papers in the collection can fit into multiple categories. Nevertheless, they serve as a useful rubric for appreciating the depth and breadth of the Rosetta Macromolecular software package.

### Protein Structure Prediction

The structural prediction of monomeric, soluble proteins is still an unsolved problem, notwithstanding notable recent advances. One important necessity in computational prediction protocols is reducing the high dimensional search space during simulations. An increasingly successful approach is the incorporation of structural restraints derived from phylogeny or low-resolution experiments–both approaches provide valuable but sparse and/or noisy information, and the challenge is to productively use these data. For example, Braun et al. demonstrate that evolutionary information on the protein fold can be discretized as residue-residue “contact maps”, and that these can be combined with iterative sampling techniques for more accurate protein structure prediction [14]. In another example, Huber and colleagues show the integration of Rosetta with sparse EPR constraints to model conformational states in a model protein [15]. One technical issue that arises with the incorporation of multiple experimentally derived restraints is that individual sets are incompatible with each other, thus requiring manual intervention from the coder. To address this problem, Porter et al. developed a computational framework that simplifies combined sampling strategies in Rosetta [16]. They then demonstrated this powerful framework on a range of modeling problems, including domain insertion and *ab initio* structure prediction with multiple sets of experimental restraints.

### Membrane Proteins

The design and modeling of membrane proteins is an emerging research area. Gray and colleagues present an integrated framework for membrane protein modeling and design [17]. In this work they showed application of the modeling framework to predict free energy changes upon mutation, high-resolution structural refinement, protein-protein docking, and assembly of symmetric protein complexes.

### Docking

A significant issue limiting the success of both protein-protein and protein-small molecule docking is the large size and ruggedness of the search space. To efficiently sample conformational space, several approximations are made in the Rosetta approach: a low resolution Monte Carlo search, typically with a coarse-grained representation of the molecules and an approximate energy function, is first performed, followed by high resolution Monte Carlo refinement with atomic resolution [18]. In spite of these approximations, sampling remains computationally inefficient. Furthermore, the energy functions used in the high-resolution step, while being more accurate than the low-resolution step, are still built for speed over accuracy, and often suffer from incorrect modeling of interactions between polar groups, and protein with the solvent. More specifically, in the Rosetta high-resolution energy function, the balance of hydrogen bonding, electrostatics and desolvation forces is a known contributor to energy function inaccuracy [8,19]. It should be noted that the limitations in scoring and sampling are related–enhanced sampling allows identification of false positive conformations, where as more accurate scoring increases ease of identification of true positive solutions by more efficient identification of more optimal basins. Several papers tackle the sampling and scoring issues in docking:

Zhang et al. show the application of replica exchange and other advance sampling techniques to increase the efficiency of Monte Carlo search during docking. Using a benchmark set of 20 protein-protein complexes, they identified an advanced sampling strategy showed better performance with equivalent computational resources. A new sampling approach was used by DeLuca et al. [20] to improve the accuracy and decrease the computational cost of the RosettaLigand docking protocol used in the prediction of protein-small molecule interactions [21]. For protein-small docking, the Karanicolas group report several significant improvements to a previously developed “ray casting” docking approach [22] used for the prediction of small molecules that disrupt protein-protein interactions [23]. Bazzoli et al. show that the use of two recent enhancements to the Rosetta energy function–explicitly including a Coulombic electrostatic term, and using a modified form of the implicit solvation potential–can markedly improve the ability to identify small-molecule inhibitors of protein-protein interactions [24].

### Protein Multispecificity Design

The design of multi-specificity of proteins is important in applications ranging from structural vaccine design, bispecific antibody therapy, and combinatorial biocatalysis. Many computational design strategies rely on genetic algorithms, which are slow and limit search space. To address this problem, the Meiler group developed a new algorithm that can find multistate minima without reliance on techniques that limit search space like a fixed backbone approximation [25].

### Scientific Benchmarks

Many of the above protocols were developed by evaluating performance against a benchmark set. Development of accessible, standard benchmarks for different end uses has the potential to increase the speed of method development, and aid reproducibility. For that reason, the Kortemme lab has developed a centralized web resource for standardized benchmark datasets (https://kortemmelab.ucsf.edu/benchmarks) [26]. This web resource includes analysis scripts, Rosetta commandlines, and tutorials for the given benchmark. There are three main sets of benchmarks in this resource: tests estimating the energetic effects upon mutation, tests for structure prediction, and ones for protein design. As a further example of the utility of benchmark sets, Ollikainen et al. developed a benchmark in order to test different protein design protocols on the re-design of enzyme substrate specificity [27]. They then showed that a protocol coupling backbone with side-chain flexibility improves prediction of sequence recovery over a competing fixed backbone approach.

Taken together, the articles in this collection highlight the utility of the Rosetta approach in tackling wide-ranging problems in biomolecular modeling and design using a common platform that allows the accessible and reproducible re-utilization of software. The common framework also provides an inherent feedback loop where new algorithms for sampling and scoring can be widely utilized and benchmarked for diverse scientific problems, in the process highlighting limitations of the approaches and areas where further developments are needed. We hope that through this collection readers will get a taste of the excitement and the unity in diversity that we enjoyed at RosettaCon 2014!
